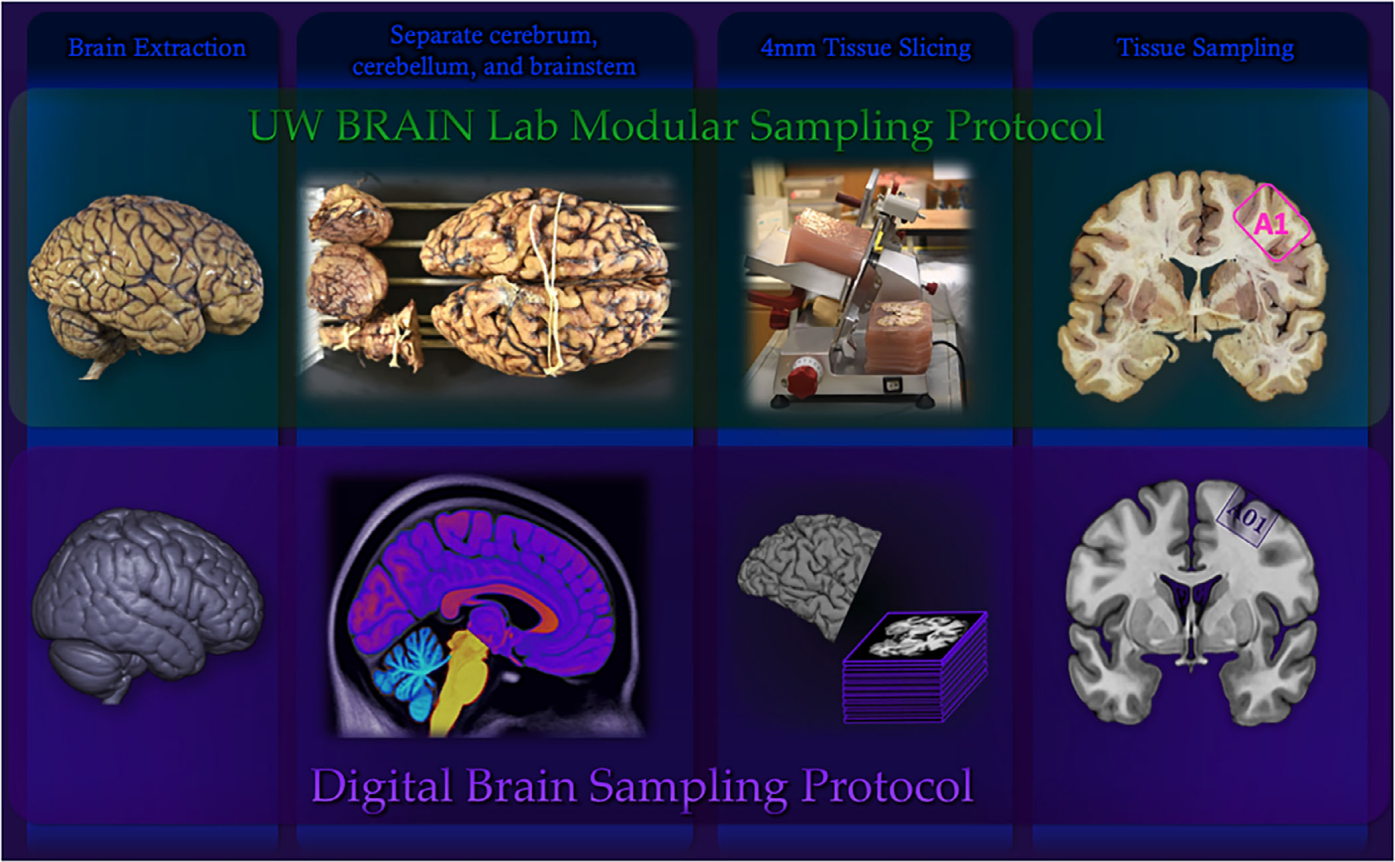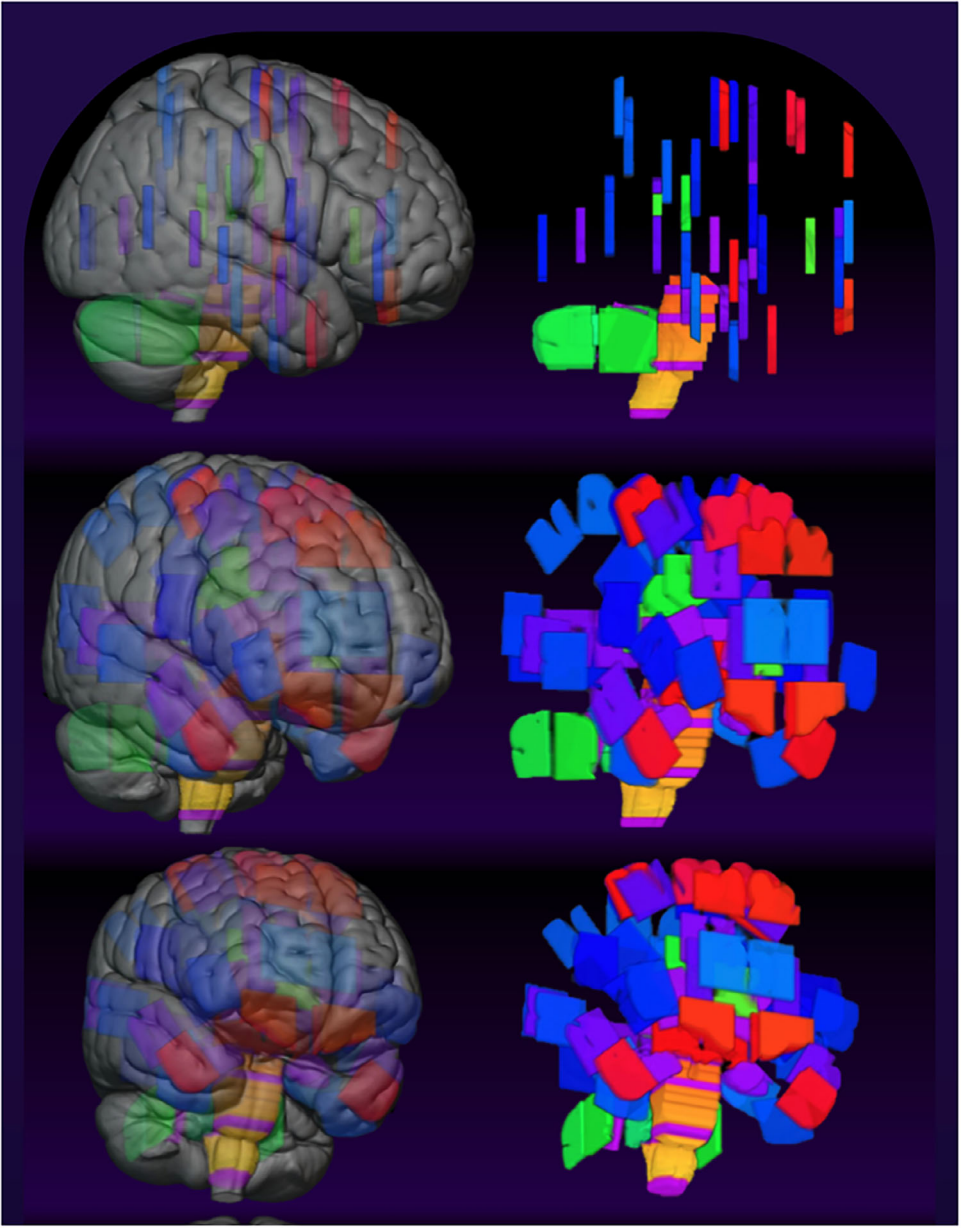# A Digital Atlas to Unlock the Potential of Brain Biorepository Tissue for Interdisciplinary Research

**DOI:** 10.1002/alz70855_107778

**Published:** 2025-12-25

**Authors:** Jason Webster, Ali Shojaie, Yiqin Alicia Shen, Tung Le, Christine MacDonald, Caitlin S Latimer, C. Dirk Keene, Thomas J Grabowski

**Affiliations:** ^1^ University of Washington, Seattle, WA, USA; ^2^ University of Washington Department of Neurological Surgery, Seattle, WA, USA; ^3^ University of Washington, School of Medicine, Seattle, WA, USA

## Abstract

**Background:**

The structural, cellular, and biomolecular research necessary for a mechanistic understanding of neuroscience relies on brain tissue collected according to a brain sampling protocol (BSP) and preserved in biorepositories. These analyses are crucial for assessing the validity of research in Alzheimer's Disease and Related Dementias. The University of Washington BioRepository and Integrated Neuropathology (BRaIN) Laboratory has developed an extensive BSP for brain preparation, sampling, characterization, and storage. The digital BSP atlas uses a neuroimaging framework to create a 3D representation of this BSP to advance the tissue request process, augment biorepository workflows, generate scientific visualizations, and provide a link to neuroimaging data and analysis methods.

**Method:**

Virtual neuropathology was performed according to the BRaIN lab BSP using the Montreal Neurological Institute and International Consortium on Brain Mapping 2009b Nonlinear Asymmetric Brain Template. Brain extraction, segmentation, and slicing were precisely replicated using FreeSurfer, FMRIB's Software Library (FSL), and custom Python/R scripts. Virtual tissue sampling was performed by the Lead Tissue Procurement Technician and a board‐certified neuropathologist in Figma, a collaborative cloud‐based platform with an intuitive dynamic interface. Data were exported in Scalable Vector Graphics (SVG) format and processed in Python to create the 3D digital BSP atlas.

**Result:**

**The Digital BSP Atlas** provides volumetric labels for all routinely collected fixed‐tissue samples in the BRaIN lab BSP and incorporates standard neuroinformatics conventions for compatibility with freely available software including FSL, MRICroGL, and FreeSurfer.

**Conclusion:**

The digital BSP atlas provides an explicit spatial representation of the BSP, enabling researchers to more efficiently locate, reference, and utilize postmortem samples. Implementing the BSP with modern neuroinformatics conventions provides a highly portable, quantitative reference that facilitates interdisciplinary collaboration, dynamic visualization, figure generation, protocol optimization, and integration with *in‐vivo* neuroimaging. Ongoing extensions of this work include the development of neuropathology sampling training software and a flexible interface to identify corresponding samples from free‐form neuroscience terms.